# Microsurgical Lymphatic Vessel Transplantation for Chronic Lymphedema: Long-Term Evaluation of Volume Reduction and Lymphatic Transport Kinetics

**DOI:** 10.3390/life15060914

**Published:** 2025-06-04

**Authors:** Wolfram Demmer, Louisa Antonie Hock, Konstantin Christoph Koban, Paul Severin Wiggenhauser, Matthias Brendel, Riccardo Giunta, Tim Nürnberger

**Affiliations:** 1 Department of Hand, Plastic and Aesthetic Surgery, Ludwig-Maximilians-University Munich, 80539 Munich, Germanytim.nuernberger@med.uni-muenchen.de (T.N.); 2 Department of Nuclear Medicine, Ludwig-Maximilians-University, 81377 Munich, Germany; 3 German Center for Neurodegenerative Diseases (DZNE), 81377 Munich, Germany; 4 Munich Cluster for Systems Neurology (SyNergy), 81377 Munich, Germany; 5 Interfaculty Center for Endocrine and Cardiovascular Disease Network Modelling and Clinical Transfer (ICONLMU), LMU Munich, 81377 Munich, Germany

**Keywords:** lymphoedema, microsurgery, lymphatic vessel transplantation, 3D imaging, lymphoscintigraphy

## Abstract

This study investigates long-term volume reduction after microsurgical autologous lymphatic vessel transplantation (LVT) in patients with chronic lymphoedema. Lymphoedema is caused by inadequate lymphatic drainage and leads to swelling, pain, and a reduced quality of life. Conservative treatments often show only limited success, which is why surgical procedures such as LVT are increasingly gaining in importance. In a retrospective long-term analysis, patients who underwent LVT between 1988 and 2010 were examined on average 21.7 years after surgery. The examination included pre- and post-operative volume measurements, which were supplemented by modern 3D body scanner analyses and lymphoscintigraphy. The results show a significant volume reduction both in the short term (*p* < 0.01) and at the follow-up examination (*p* = 0.04). There was no significant difference between manual volumetry with circumferential measurements and 3D volumetry (*p* = 0.775). The improvement in lymph transport capacity was considerable (*p* = 0.078). This study provides valuable insights for the further development of lymphatic surgery. While preferred surgical methods change over time, this study demonstrates that LVT can make a decisive contribution to improving the quality of life of lymphedema patients.

## 1. Introduction

The lymphatic system is the second most important transport system in the human body after the blood circulation. It performs essential functions in the transport of fluid, proteins, antigens, lymphocytes, chylomicrons, and other cells of the immune system. It also transports pathogens such as bacteria and foreign bodies to the lymph nodes, where they are presented to the immune system [1,2,3,4]. In healthy people, the transport capacity of the lymphatic vessels is ten times higher than the actual lymph load required by the tissue [5]. This enables effective drainage of the lymph fluid, prevents congestion and accumulation, and, thus, protects against the development of lymphoedema [2,3].

However, when the transport capacity of the lymph vessels for lymph drainage from the tissue is not sufficient to meet the demand, lymphoedema occurs. This can have both primary and secondary causes [6,7]. Primary lymphoedema occurs much less frequently than secondary lymphoedema. The prevalence of secondary lymphoedema is around 1/1000, while that of primary lymphoedema is only around 1/100,000 [8,9]. While primary lymphoedema is due to congenital maldevelopment of the lymphatic system, secondary lymphoedema is often caused by the removal of local lymph nodes or the destruction of lymph vessels [10]. They are a frequent complication after oncological operations, trauma, radiotherapy, or infections, particularly in industrialized countries, and mainly affect the extremities. If left untreated, lymphoedema progresses through successive stages (I–III) and becomes an irreversible chronic condition associated with fibrosclerotic tissue changes, pathological fat and connective tissue proliferation, and an increased predisposition to acute skin infections such as erysipelas [11,12,13]. Patients with chronic lymphoedema suffer from considerable pain, pressure, tension, and daily volume fluctuations. The condition also causes severe biopsychosocial limitations and a significantly lower quality of life than in healthy people [14,15,16,17]. Swollen limbs are both aesthetically disturbing and a hindrance to daily activities [18]. As a result, the patients are also likely to suffer from psychological disorders such as anxiety and depression [19].

It is estimated that from around 140 to 200 million people worldwide are affected by lymphoedema [20]. The prevalence in Germany is estimated at around 1.5–2% [21]. Despite advances in the treatment of underlying oncological diseases, secondary lymphoedema remains a significant and distressing complication of cancer treatment, in particular [22]. This results in a particularly strong interest in investigating effective treatment options for this disease.

**Figure 1:**
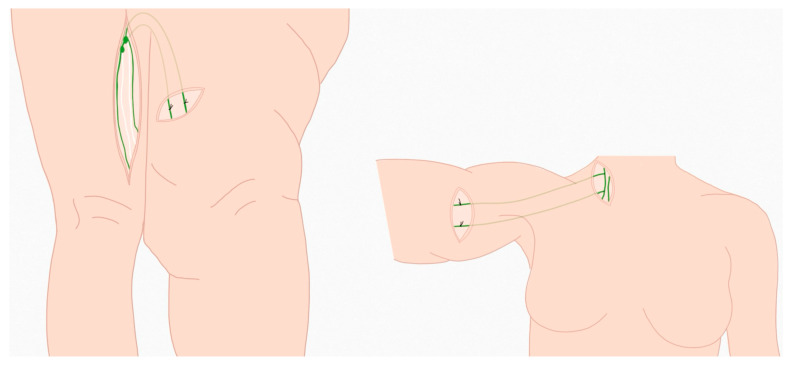
Schematic representation of the microsurgical autologous lymphatic vessel transplantation. The lymphatic vessel to be taken is dissected. During the interposition to the other leg (**left**), the vessel remains connected to the lymph collectors of the right leg and is guided through an incracutaneous channel to the region with lymphoedema and fixed there microsurgically. In the case of interposition to the arm (**right**), the entire vessel is removed, microsurgically fixed to the arm, guided intracutaneously to the neck lymph collectors, and connected to these by an end-to-side anastomosis.

Conservative therapy primarily includes measures such as decongestive compression therapy, manual lymphatic drainage, and skin care. However, these methods are time-consuming, require a high level of compliance, and often do not lead to complete relief of the symptoms [9]. Surgical treatment is required for refractory or advanced lymphoedema [23]. Various microsurgical therapies such as lymphovenous anastomosis (LVA), vascularized lymph node transfer (VLNT), liposuction, or debulking surgery are currently recommended [9,20,24,25,26]. Autologous lymph vessel transplantation, the microsurgical transfer of individual lymph vessels to bridge localized lymph congestion caused by damaged tissue, is an established method in lymphatic surgery first performed in 1980 [27,28,29,30,31]. A lymphatic vessel is usually taken as a graft from the thigh or groin. The surgeon searches for a suitable lymph vessel that is healthy and functional. This is usually determined beforehand using lymphatic vessel scintigraphy and stained intraoperatively using fluorescent dye. By making a longer incision in the skin, one or more lymph vessels up to 30 cm in length are freely dissected and removed. Pedicled grafts can also be removed from the lower extremity, in which a connection to the original lymph nodes is retained. Tissue-sparing work is carried out to ensure that lymphatic drainage continues at the site. The transplant is guided through a subcutaneous tunnel to the target region, usually from the thigh to the leg or arm. The grafts are then microsurgically connected to the existing lymphatic system of the target region using lympho-lymphatic, lymo-venous, or lympho-lymphonoduary end-to-side anastomoses [27,28] (Figure 1). The surgeon checks the patency of the new connections using fluorescent dye to ensure that the lymph can drain effectively in the transplant [23,32]. For this purpose, patent blue was used in the past. Nowadays, indocyanine green is used since it is more effective for the intraoperative visualization of lymphatic vessels than patent blue [32]. Postoperatively, the permeability is checked by lymphatic drainage scintigraphy [27,28].

A comparison of permeability for autologous lymphatic vessel transplants (LVT), allogeneic lymphatic vessel transplants, autologous vein transplants, and expanded polytetrafluoroethylene (ePTFE) microprostheses found autologous lymphatic vessel transplants to be the superior surgical treatment option in chronic lymphedema [23]. Earlier studies showed significant improvements in lymph transport, a reduction in swelling, and an increase in quality of life [33,34]. However, long-term data evaluating the benefits of this method over a period of more than ten years is still lacking.

The objective of this study is to analyze the long-term results of microsurgical autologous lymphatic vessel transplantation (LVT). Building on the results of previous work, this study aims to provide new, long-term findings and supplement existing data.

A particular focus is on innovative approaches to the diagnosis of lymphoedema. In addition to the retrospective evaluation of pre- and postoperative findings, a comprehensive clinical follow-up examination was carried out. This included an objective evaluation of the success of the treatment by means of circumference and volume measurements, supplemented by a modern digital procedure—the Vectra^®^ Whole Body 3D Scanner (Canfield Scientific, Parsippany, NJ, USA). In this context, this study examines the extent to which three-dimensional volumetry can replace the conventional manual measurement method in the future. To assess the functional effectiveness, the lymph transport capacity was also examined using lymph vessel scintigraphy. Previously published data from this study cohort showed that patients’ health-related quality of life (HRQoL) improved significantly in the long term after LVT [35]. Both HRQoL and 3D volumetry are becoming increasingly important as supplementary parameters in the evaluation of treatment success.

This study thus provides important insights into the efficacy and safety of the microsurgical technique of LVT and contributes to the further development of lymphatic surgery, with the aim of alleviating the suffering caused by comorbidities in many lymphoedema patients in the long term.

## 2. Materials and Methods

This retrospective study assesses the volume reduction and changes in health-related quality of life in patients diagnosed with lymphedema of both lower and upper limbs who have been treated by microsurgical autologous LVT. Patients diagnosed with lymphedema were recruited from the Department of Hand, Plastic, and Aesthetic Surgery of the LMU Clinic Munich.

Inclusion criteria of patients involved microsurgical therapy of a manifested lymphoedema by LVT by the Department of Hand, Plastic, and Aesthetic Surgery of the LMU Clinic Munich or its precursor organization, the section of plastic surgery, hand surgery, microsurgery, and surgical clinic Großhadern between 1 January 1983 and 31 October 2010. All patients aged 18 years and older who agreed to participate were included.

**Figure 2:**
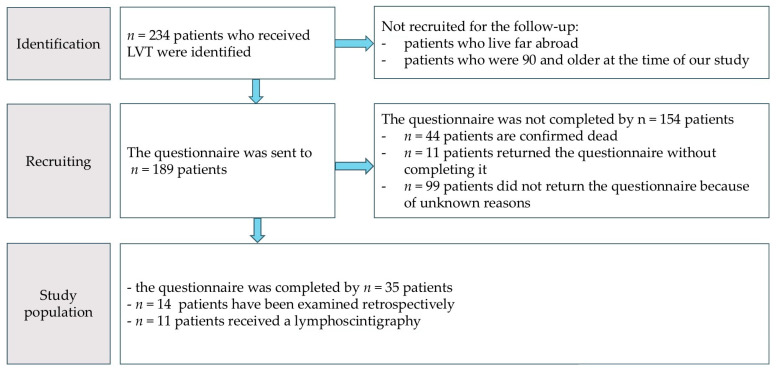
Flowchart of the study process.

Exclusion criteria encompassed an unbreachable language barrier or the patient’s refusal to participate in this study. Ethical approval was obtained from the Ethics Committee of the LMU Munich prior to the commencement of examinations (Project.: 23-0147, Date: 23 August 2023) (Figure 2).

Eligible participants were identified via the hospital’s electronic records system and by manually reviewing older paper files. They were contacted by mail or email when available. A total of 189 questionnaires were sent to patients, but the long latency period since the surgery resulted in a comparatively high dropout rate of 80%, leaving 35 participants in the final study population.

### 2.1. Assessment Instruments

#### 2.1.1. Manual Volumetry

The patients were called in for clinical follow-up examinations. Manual circumferential limb measurements were carried out first. This method has proven to be reliable for determining the volume of lymphoedema, especially when carried out by trained personnel [36,37]. Measurements were carried out in the same way as preoperatively, to ensure comparability. The circumferences of the extremities were measured manually every 4 cm, and the volumes were calculated using the formula *Vol* = (*C*1^2^ + *C*2^2^ + …*Cn*^2^)/*π*. This method was used to determine the volume of the healthy limb and the limb affected by the lymphoedema. The relative difference in volume between both extremities was then calculated. This provides a good indication of the extent of the lymphoedema in clinical practice and is also suitable as a parameter for progression.

#### 2.1.2. Digital 3D Volumetry

In addition, we tested the volume measurement using a digital radiation-free method, the Vectra^®^ Whole Body 3D Scanner. It creates a high-resolution three-dimensional image of the patient using multiple cameras [38]. The reconstructed 3D model was then imported into the related Sculptor^®^ software (Dassault Systemes Deutschland GmbH, Stuttgart, Germany) to calculate the volumes of the extremities. The measurement was performed aligned in the XZ plane to ensure an exact calculation [39,40]. Similarly to the circumferential disc method, the total volume was calculated without food or hand volume and up to the cranial marking in the gluteal fold or the medial marking in the axillary fold. The areas used for measurement with the 3D scanner corresponded exactly to the areas measured by manual volumetry, as the markings placed during the manual measurement served as an orientation aid. As the Vectra scanner is a relatively new method that has only been used to perform volumetry for a few years, there are no preoperative images that have been taken with this method. The three-dimensional digital volumetry can therefore only be compared with the results of manual circumferential volumetry obtained today and not with preoperative measurements.

#### 2.1.3. Lymphoscintigraphy

A lymph vessel scintigraphy of the affected extremity was performed. They were compared to the pre- and postoperative results already obtained in the patients included in our study at our clinic, ensuring good comparability. Lymphoscintigraphy is considered the gold standard in the diagnosis of lymphoedema [9,41]. This functional imaging makes it possible to assess lymph transport and can detect pathological changes at an early stage (stage 0) that are not yet clinically visible. Lymphoscintigraphy provides precise information on lymphatic drainage and is crucial for classification and treatment planning [42,43,44,45]. The transport index was calculated using the findings of the preoperative lymphatic scintigraphies and the findings of the scintigraphies performed as part of this study. The TI was then calculated using the formula *TI* = *temporal distribution of the tracer* + *spatial distribution of the tracer* + *lymph node appearance time* + *lymph node visualisation* + *visualisation of the lymph collectors*. Each item can achieve 0–9 points, with 0 points correlating with a perfect physiological lymphatic drainage and 9 points with a pathological lymph congestion. A maximum of 45 points can be achieved [46].

Statistical analyses were performed for the entire patient cohort and for patients with lower and upper limb lymphedema, respectively. The demographic parameters were correlated with the data from the SF-12 questionnaires and the collected edema measurements, pre- and postoperative lymphatic scintigraphies from a data registry, such as the underlying disease, pre- and postoperative manual edema measurements, pre- and postoperative lymphatic scintigraphies, previous treatment, and medical history. The results were analyzed using IBM^®^ SPSS^®^ 29 Statistics according to standard statistical procedures. A significant correlation with a *p*-value < 0.05 was assumed.

## 3. Results

The patients included in our study had undergone LVT on average 21.7 ± 4.9 years ago. Of the respondents who completed the questionnaire, 17 had upper extremity lymphedema and 18 had lower extremity lymphedema. Six patients had primary lymphedema, while 29 had secondary lymphedema. Of the 35 respondents, 31 were female and 4 were male. Of these patients, some received a manual follow-up examination, with 14 patients receiving a physical examination with manual circumferential volume measurement and Vectra WB 360 Scan. A total of 11 patients also received a lymphatic scintigraphy. All included patients had received at least six months of unsuccessful conservative therapy prior to surgery, with a mean duration of 4.8 ± 3.1 years.

To assess the representativeness of the cohort, a chi-square test for independence was performed. In this analysis, the characteristics of the patients included in the follow-up were compared with those of the 154 patients who did not participate in this study. The analysis was performed using variables such as lymphoedema type, age at the time of surgery, age at study participation, and gender distribution. No significant differences were found between the two groups for these variables. For example, the *p*-value for gender distribution was 0.87, which reflects the high proportion of female patients and is consistent with the higher prevalence of lymphoedema in women in Central Europe, which is estimated to be 4.5–6.1 times more common than in men 36. The *p*-value for the type of lymphedema was 0.651. A Mann–Whitney U test was performed to compare age at surgery and age at study participation. The analysis showed no significant differences between the participants and the patients who did not take part in this study. Of the original 189 patients, 44 had already died at the time of this study. Although the causes of death could not be determined in all cases, it can be assumed that most of them died from natural, age-related causes or from an underlying disease. This is also due to the fact that many of the patients included in this study are long-term cancer patients.

Overall, it can be concluded that the cohort of participants was representative of the larger group of patients who had also undergone LVT and did not participate in our study. No significant differences were found in the variables analyzed, which ensures the validity of the results derived from the study cohort.

### 3.1. Volume Reduction

The volume of the affected extremities was determined using the circumferential measurement method at both the preoperative and several postoperative stages to quantitatively assess the effect of the surgical intervention on tissue swelling. Based on these measurements, the relative volume difference between the healthy and the operated limb was calculated, reflecting the degree of volume strain.

In the preoperative state, the relative volume difference averaged 24.3 ± 12.8 percentage points (mean ± standard deviation), which represents the initial state of a significant increase in volume in the affected extremity.

**Figure 3:**
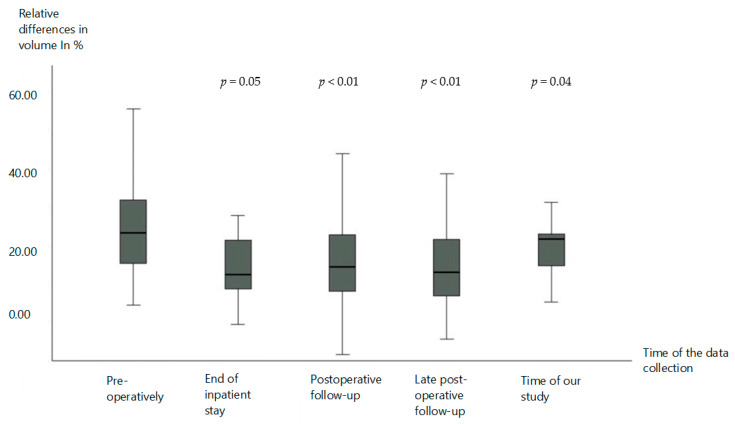
Changes in the volume of the lymphoedema. Data are represented as from the 25th to 75th percentiles (boxes) with the median (line in the box). The X-axis shows the time of the data collection, respectively, preoperatively, and postoperatively at the end of the inpatient stay, at the time of the postoperative follow-up examination, at the time of a late postoperative follow-up examination, and at the time of our study. The Y-axis shows the relative differences in volume. The *p*-value was determined using the *t*-test for independent samples.

At the end of the inpatient stay, a significant reduction in the relative volume difference was observed, which averaged 13.8 ± 12.1 percentage points. This corresponds to an average improvement of 10.5 percentage points. Statistically, this change was evaluated with a *p*-value of 0.05, which demonstrates a moderately significant reduction (Figure 3).

Postoperative follow-up examinations after an average of 7.8 ± 12.4 months showed a further improvement in the relative volume difference, which amounted to an average of 15.6 ± 11.0 percentage points at this time. This corresponds to a reduction of 

8.7 percentage points compared to the initial value. The statistical analysis resulted in a *p*-value of <0.01, which indicates a significant improvement in the volume load (Figure 3).

In a longer-term, late postoperative follow-up examination after 6.1 ± 6.6 years, a relative volume difference of 15.1 ± 10.5 percentage points was determined. This also shows an improvement of 9.2 percentage points on average compared to the preoperative state. With a *p*-value of <0.01, this change was also evaluated as statistically significant (Figure 3).

The retrospective analysis of our study cohort, which occurred, on average, 21.7 ± 4.9 years after surgery, showed an average relative volume difference of 20.6 ± 8.0 percentage points. This corresponds to an average reduction in the difference of 3.7 percentage points compared to the preoperative state. This difference was classified as moderately significant with a *p*-value of 0.04 (Figure 3).

### 3.2. New Diagnostics for the Measurement of Volumes

In order to compare the classical volume determination by circumferential measurements and the modern volumetric analysis using the Vectra 360 Whole Body Scanner in patients with lymphedema, they were applied successively to the same patients to analyze the determined volumes and the time required for each method.

**Figure 4:**
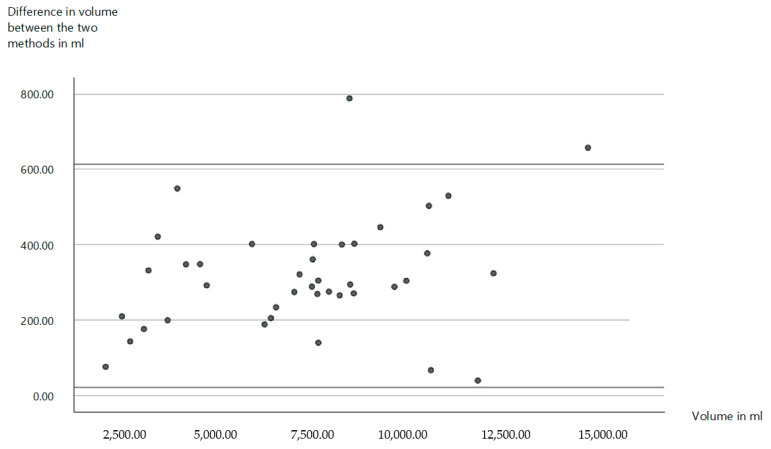
Distribution of volume differences of both measurement methods as a function of the measured volume. Each point represents an individual data set from the cohort. The X-axis shows the volume measured by the Vectra WB 360 Scanner and the Sculptor Software. The Y-axis shows the differences in volume between the methods. The inserted vertical lines correspond to the standard deviation from the mean value, respectively, 317.18 ± 151.5.

In terms of measured volumes, the circumferential method showed an average volume of 9012.8 ± 2234.5 mL (mean ± standard deviation) for the lower limb and 3838.7 ± 1233.4 mL (mean ± standard deviation) for the upper limb. Using the Vectra 360 Whole Body Scanner and the Sculptor software, however, a volume of 8669.6 ± 2085.6 mL (mean ± standard deviation) was determined for the lower extremity and 3546.3 ± 1079.8 mL (mean ± standard deviation) for the upper extremity. This corresponds to a total difference of 317.8 ± 151.5 mL (mean ± standard deviation) or 4.4 ± 2.1% (mean ± standard deviation) between the two measurement methods. Statistically, a *p*-value of 0.775 was obtained, indicating no significant difference between the two methods (Figure 4).

### 3.3. Lymphoscintigraphy

**Figure 5:**
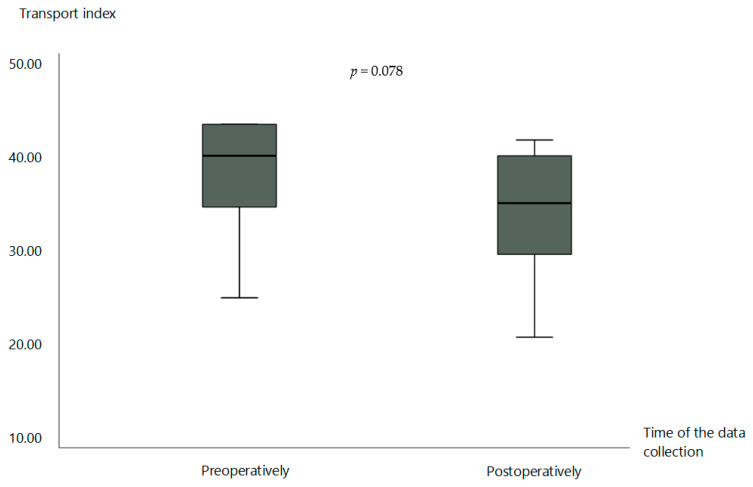
Changes in the TI of the lymphatic grafts. Data are represented as from the 25th to 75th percentiles (boxes) with the median (line in the box). The X-axis shows the time of the data collection, respectively, before and after the LVT. The Y-axis shows the calculated score of the TI. The *p*-value was determined using the *t*-test for independent samples.

**Figure 6:**
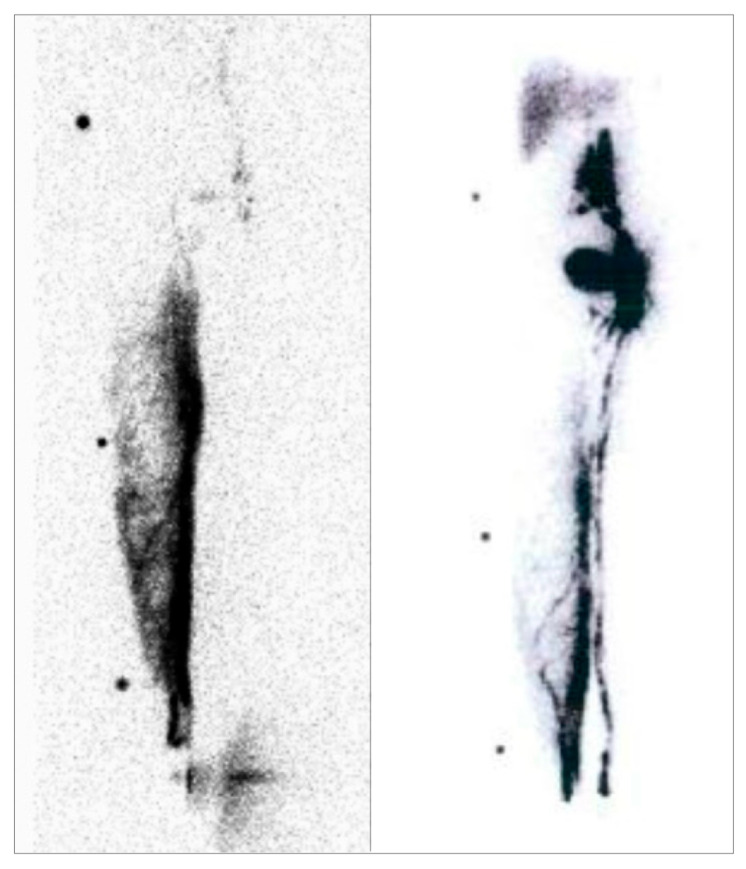
Lymph vessel scintigraphy of the affected right leg preoperatively (**left**) and postoperatively (**right**).

In the preoperative state, the average transport index was 34.2 ± 7.4 points (mean ± standard deviation), which indicates a clearly limited transport capacity of the affected lymphatic vessels. After the operation, a reduction in the transport index was observed, with the value of the graft averaging 30.0 ± 8.2 points (mean ± standard deviation). This reduction represents an improvement in the transport index by an average of 4.2 points (*p* = 0.078). The data indicate a considerable but not quite statistically significant improvement in lymphatic transport kinetics (Figure 5). Figure 6 shows an example of lymphoscintigraphy performed preoperatively and during postoperative follow-up.

## 4. Discussion

The results show a clear reduction in the relative increase in volume of the respective limb over the course of the operation. The long-term results after an average of 21.7 ± 4.9 years show a permanent reduction, which most likely resulted from the lymph vessel transplantation. We have analyzed the results over the course of the therapy. It is striking that the relative volume reduction was greatest at the end of the inpatient stay after the operation. This can probably best be explained by the fact that manual lymphatic drainage and physical compression therapy with compression stockings were carried out permanently and by trained medical staff during the inpatient’s stay. In addition, patients stand and sit less during this period than in everyday life, which, in turn, also results in less severe lymph congestion and therefore less pronounced oedema [9,47,48].

Within the scope of our follow-up examinations, the relative volume differences were the least pronounced. This implies that the volume reduction achieved by LVT decreases again in the course of the postoperative course. However, a significant volume reduction of 20.6 ± 8.0 percentage points can still be demonstrated after 21.7 ± 4.9 years postoperatively. However, the relative decline in the postoperative surgical success over the years could be explained by the age-related decrease in lymphatic transport. The loss of matrix proteins and smooth muscle cells leads to a limited pumping activity and a reduced ability to maintain tissue fluid homeostasis with age [49].

An improvement in TI after the LVT could already be demonstrated [1,46,50,51]. Moreover, lymph vessel scintigraphy has also shown that LVT does not harm the donor limb and that lymphatic drainage can still be visualized there by scintigraphy [44]. Our results are consistent with these previous findings and indicate a long-lasting postoperative improvement in transport kinetics within the lymphoscintigraphy. The clinical findings correlate with what has been observed clinically for a long time and what we have already been able to prove within this study. Namely, a significant improvement in HRQoL, particularly with regard to the feeling of pressure and tension, pain, volume fluctuations during the course of the day, and the frequency of manual lymphatic drainage to alleviate the symptoms [35].

**Figure 7:**
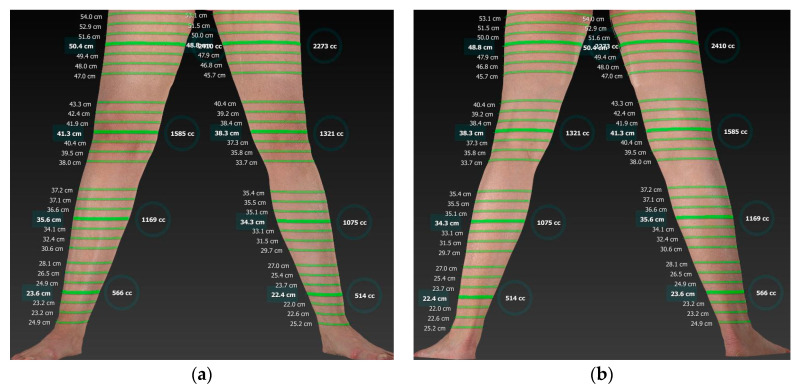
A 59-year-old patient with lymphoedema of the right leg after lymphadenectomy within the surgical treatment of cervical carcinoma treated by LVT with lymphatic vessels from the left thigh. The circumference and volume measurements of the affected right leg and the left leg, as the donor side, are depicted 21 years after LVT as three-dimensional images taken with the Vectra 360 Whole Body Scanner from the front (**a**) and from behind (**b**).

**Figure 8:**
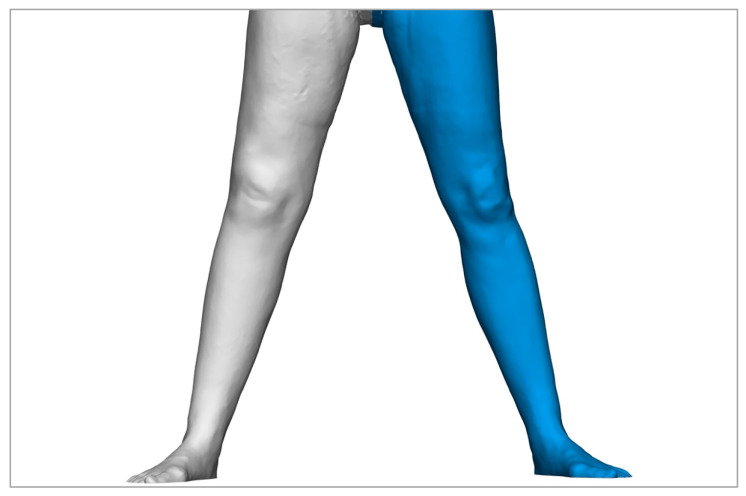
A 59-year-old patient with lymphoedema of the right leg after lymphadenectomy within the surgical treatment of cervical carcinoma, treated by LVT with lymphatic vessels from the left thigh. Three-dimensional image taken with the Vectra 360 WB Scanner showing the healthy leg colored blue and the affected leg in grey, 21 years after LVT.

**Figure 9:**
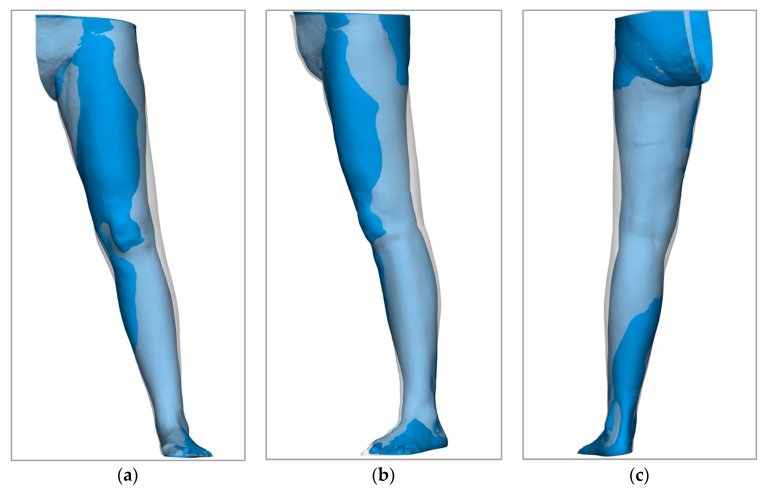
A 59-year-old patient with lymphoedema of the right leg after lymphadenectomy, within the surgical treatment for cervical carcinoma treated by LVT with lymphatic vessels from the left thigh. These three-dimensional images taken with the Vectra 360 WB Scanner show the affected leg (in grey) and the healthy leg (in blue) overlaid from the front (**a**), the side (**b**), and the back (**c**).

The choice of method for determining the volume of extremities plays a central role in the diagnosis and treatment planning of lymphoedema. However, the methods considered here, circumferential measurement and volumetric measurement using Vectra 3D technology, as well as the water displacement method, which we do not use, differ significantly in their practicability, accuracy, and applicability [9]. The water displacement method has traditionally been regarded as the gold standard for volume measurement, as it determines volume differences very accurately. However, it has proven to be difficult to apply in clinical practice. This is due to the high time expenditure and difficulties in handling. These factors severely limit the use of the water displacement method in everyday clinical practice and make it unattractive for many settings [22,52]. Circumference measurement, on the other hand, is often favored because it is easy to use and quick to perform. However, it has been shown that the circumference measurement overestimates the actual volume of the extremities. We were able to confirm this in comparison to Vectra technology with an average deviation of 317 ± 151 mL. Previous studies were also able to demonstrate even greater deviations in some cases [53]. This systematic overestimation could lead to incorrect assessments of the severity of oedema in practice. In addition, the circumference measurement is more susceptible to individual errors. On the one hand, repeated measurements by different examiners. Interrater reliability is limited, which can impair the comparability and reproducibility of measurements. The stability and reproducibility of three-dimensional methods, such as the Vectra WB 360 scanner, for determining the volume of lymphoedema have already been demonstrated in the past. Volumetric measurement using Vectra 3D technology, therefore, represents a more precise and reproducible alternative [53,54,55]. The electronic storage of the data increases traceability and minimizes inter-individual differences in performance. In addition, 3D technologies offer the decisive advantage of differentiating between homogeneous and inhomogeneous volume accumulations [53]. This possibility is lacking in both the circumference measurement and the water displacement method and allows a more precise assessment of the oedema, which could be particularly advantageous in surgical planning. These advantages are clearly illustrated by Figure 5, Figure 7, Figure 8 and Figure 9. However, there are also disadvantages associated with the 3D technology. The high acquisition costs and the demanding operation are major barriers, especially in facilities with a limited budget or without specialized staff. In addition, artificial errors in volume determination can occur due to particularly pronounced skin folds, crypts, or other unevenness of the skin surface [53]. Another problem is that hands and feet, which are often affected by oedema, cannot currently be recorded. In addition, part of the thigh or arm is lost due to the cranial or medial boundary at the gluteal and axillary fold, which makes it difficult to analyze the entire limb. However, it should be borne in mind that the same applies to the cone and water displacement method. Overall, we can therefore emphasize the previously demonstrated suitability of three-dimensional volumetric methods for the diagnosis and monitoring of lymphoedema [56].

Improvement of lymphatic transport kinetics was considerably enhanced by LVT. The limited size of the study subgroup undergoing a control lymphoscintigraphy can be assumed as the reason why this effect could not be statistically proven.

## 5. Conclusions and Implications for Further Research

The results of this retrospective study confirm the effectiveness of LVT in the surgical treatment of chronic lymphedema in terms of volume reduction and improvement of lymphatic transport kinetics. Future research is still needed to evaluate the long-term results of LVT with a larger cohort of patients, for example, as part of a meta-analysis. The results could also be compared with a control cohort in order to obtain an even more meaningful picture. Future research should also compare the long-term results of LVT with other surgical and conservative treatment options. In the future, it may also be important to investigate quality of life after surgical treatment of lymphoedema, not only in relation to disease-specific factors, but also to non-specific factors that may contribute to treatment success. In order to optimize the use of 3D technologies in the field of lymphoedema and lipedema diagnostics, a specific tool to improve the detection of hands and feet and include a more precise definition of border areas is needed. Despite its weaknesses, circumferential measurement is currently the most practicable and cheapest method in everyday clinical practice, while Vectra technology is a promising tool for specialized applications due to its precision and versatility. For practical reasons, the water displacement method is only of secondary importance. However, further development and adaptation of volumetry using 3D technology could have the potential to improve the diagnosis and treatment of lymphoedema and lipedema in the long term. Prospective studies with constant and simultaneous monitoring of lymphedema patients treated by different therapies, in which volume changes are also correlated with HRQoL, are needed. This further research will help evaluate the outcome of different therapy options and diagnostic tools. Through this, the best treatment options can be identified in terms of objective and subjective parameters, ultimately helping physicians make informed treatment recommendations in the future.

## Data Availability

The original data presented in this study are openly available in PubMed at the DOI.

## References

[1] Null M., Arbor T.C., Agarwal M. (2024). Anatomy, Lymphatic System. StatPearls.

[2] Ellis S. (2006). Structure and function of the lymphatic system: An overview. Br. J. Community Nurs..

[3] Ozdowski L., Gupta V. (2024). Physiology, Lymphatic System. StatPearls.

[4] Mehrara B.J., Radtke A.J., Randolph G.J., Wachter B.T., Greenwel P., Rovira I.I., Galis Z.S., Muratoglu S.C. (2023). The emerging importance of lymphatics in health and disease: An NIH workshop report. J. Clin. Investig..

[5] Casley-Smith J.R., Földi M. (1987). Lymphangiology.

[6] Sung C., Wang S., Hsu J., Yu R., Wong A.K. (2022). Current Understanding of Pathological Mechanisms of Lymphedema. Adv. Wound Care.

[7] Baumeister R.G.H. (2017). Management of Lymphedema. Dtsch. Med. Wochenschr..

[8] Brix B., Sery O., Onorato A., Ure C., Roessler A., Goswami N. (2021). Biology of Lymphedema. Biology.

[9] Flores T., Bergmeister K.D., Staudenherz A., Pieber K., Schrögendorfer K.F. (2021). Diagnosis, prevention and therapy of lymphedema. Wien. Klin. Wochenschr..

[10] Sleigh B.C., Manna B. (2024). Lymphedema. StatPearls.

[11] Zvonik M., Földi E., Felmerer G. (2011). The effects of reduction operation with genital lymphedema on the frequency of erysipelas and the quality of life. Lymphology.

[12] Földi E., Baumeister R.G.H., Bräutigam P., Tiedjen K.U. (1998). Zur Diagnostik und Therapie des Lymphödems. Dtsch. Arztebl. International.

[13] Földi E., Földi M., Kubik S. (2010). Földi’s Textbook of Lymphology.

[14] Jäger G., Döller W., Roth R. (2006). Quality-of-life and body image impairments in patients with lymphedema. Lymphology.

[15] Moffatt C.J., Franks P.J., Doherty D.C., Williams A.F., Badger C., Jeffs E., Bosanquet N., Mortimer P.S. (2003). Lymphoedema: An underestimated health problem. QJM.

[16] Mercier G., Pastor J., Moffatt C., Franks P., Quéré I. (2019). LIMPRINT: Health-Related Quality of Life in Adult Patients with Chronic Edema. Lymphat. Res. Biol..

[17] Ridner S.H. (2009). The psycho-social impact of lymphedema. Lymphat. Res. Biol..

[18] Ton T.G., Mackenzie C., Molyneux D.H. (2015). The burden of mental health in lymphatic filariasis. Infect. Dis. Poverty.

[19] Son A., O’Donnell T.F., Izhakoff J., Gaebler J.A., Niecko T., Iafrati M.A. (2019). Lymphedema-associated comorbidities and treatment gap. J. Vasc. Surg. Venous Lymphat. Disord..

[20] Gallagher K., Marulanda K., Gray S. (2018). Surgical Intervention for Lymphedema. Surg. Oncol. Clin. N. Am..

[21] Rabe E., Pannier-Fischer F., Bromen K., Schuldt K., Stang A., Poncar C., Wittenhorst M., Bock E., Weber S., Jockel K.H. (2003). Bonn Vein Study by the German Society of Phlebology. Phlebologie.

[22] Anuszkiewicz K., Jankau J., Kur M. (2023). What do we know about treating breast-cancer-related lymphedema? Review of the current knowledge about therapeutic options. Breast Cancer.

[23] Baumeister R.G.H. (2016). Reconstructive Lymph Vascular Surgery.

[24] Garza R., Skoracki R., Hock K., Povoski S.P. (2017). A comprehensive overview on the surgical management of secondary lymphedema of the upper and lower extremities related to prior oncologic therapies. BMC Cancer.

[25] Schaverien M.V., Coroneos C.J. (2019). Surgical Treatment of Lymphedema. Plast. Reconstr. Surg..

[26] Sanka S.A., Chryssofos S., Anolik R.A., Sacks J.M. (2025). Advances in surgical management of chronic lymphedema: Current strategies and future directions. Med. Oncol..

[27] Baumeister R.G., Frick A. (2003). The microsurgical lymph vessel transplantation. Handchir. Mikrochir. Plast. Chir..

[28] Baumeister R.G., Mayo W., Notohamiprodjo M., Wallmichrath J., Springer S., Frick A. (2016). Microsurgical Lymphatic Vessel Transplantation. J. Reconstr. Microsurg..

[29] Felmerer G., Sattler T., Lohrmann C., Tobbia D. (2012). Treatment of various secondary lymphedemas by microsurgical lymph vessel transplantation. Microsurgery.

[30] Baumeister R.G., Seifert J., Hahn D. (1981). Autotransplantation of lymphatic vessels. Lancet.

[31] Baumeister R.G.H., Seifert J., Wiebecke B., Heberer G., Feifel K., Meßmer G. (1980). Transplantation of lymph vessels on rats as well as a first therapeutic application on the experimental lymphedema of the dog. Proceedings of the 97th Congress of the German Society of Surgery, Munich, Germany, 14–19 May 1980.

[32] Hackethal A., Hirschburger M., Eicker S.O., Mucke T., Lindner C., Buchweitz O. (2018). Role of Indocyanine Green in Fluorescence Imaging with Near-Infrared Light to Identify Sentinel Lymph Nodes, Lymphatic Vessels and Pathways Prior to Surgery—A Critical Evaluation of Options. Geburtshilfe Frauenheilkd..

[33] Springer S., Koller M., Baumeister R.G., Frick A. (2011). Changes in quality of life of patients with lymphedema after lymphatic vessel transplantation. Lymphology.

[34] Baumeister R.G., Siuda S. (1990). Treatment of lymphedemas by microsurgical lymphatic grafting: What is proved?. Plast. Reconstr. Surg..

[35] Hock L.A., Nurnberger T., Koban K.C., Wiggenhauser P.S., Giunta R., Demmer W. (2024). Quality of Life in Lymphedema Patients Treated by Microsurgical Lymphatic Vessel Transplantation-A Long-Term Follow-Up. Life.

[36] Farina G., Galli M., Borsari L., Aliverti A., Paraskevopoulos I.T., LoMauro A. (2024). Limb Volume Measurements: A Comparison of Circumferential Techniques and Optoelectronic Systems against Water Displacement. Bioengineering.

[37] Armer J.M., Ballman K.V., McCall L., Armer N.C., Sun Y., Udmuangpia T., Hunt K.K., Mittendorf E.A., Byrd D.R., Julian T.B. (2019). Lymphedema symptoms and limb measurement changes in breast cancer survivors treated with neoadjuvant chemotherapy and axillary dissection: Results of American College of Surgeons Oncology Group (ACOSOG) Z1071 (Alliance) substudy. Support. Care Cancer.

[38] De Stefani A., Barone M., Hatami Alamdari S., Barjami A., Baciliero U., Apolloni F., Gracco A., Bruno G. (2022). Validation of Vectra 3D Imaging Systems: A Review. Int. J. Environ. Res. Public Health.

[39] Hameeteman M., Verhulst A.C., Vreeken R.D., Maal T.J., Ulrich D.J. (2016). 3D stereophotogrammetry in upper-extremity lymphedema: An accurate diagnostic method. J. Plast. Reconstr. Aesthetic Surg..

[40] Erends M., van der Aa T., de Grzymala A.P., van der Hulst R. (2014). Validity and reliability of three-dimensional imaging for measuring the volume of the arm. Lymphat. Res. Biol..

[41] Hassanein A.H., Maclellan R.A., Grant F.D., Greene A.K. (2017). Diagnostic Accuracy of Lymphoscintigraphy for Lymphedema and Analysis of False-Negative Tests. Plast. Reconstr. Surg. Glob. Open.

[42] Weissleder H., Weissleder R. (1988). Lymphedema: Evaluation of qualitative and quantitative lymphoscintigraphy in 238 patients. Radiology.

[43] Weiss M., Baumeister R.G., Tatsch K., Hahn K. (1996). Lymphoscintigraphy for non-invasive long term follow-up of functional outcome in patients with autologous lymph vessel transplantation. Nuklearmedizin.

[44] Weiss M., Baumeister R.G., Frick A., Wallmichrath J., Bartenstein P., Rominger A. (2015). Lymphedema of the upper limb: Evaluation of the functional outcome by dynamic imaging of lymph kinetics after autologous lymph vessel transplantation. Clin. Nucl. Med..

[45] Pappalardo M., Cheng M.H. (2020). Lymphoscintigraphy for the diagnosis of extremity lymphedema: Current controversies regarding protocol, interpretation, and clinical application. J. Surg. Oncol..

[46] Kleinhans E., Baumeister R.G., Hahn D., Siuda S., Bull U., Moser E. (1985). Evaluation of transport kinetics in lymphoscintigraphy: Follow-up study in patients with transplanted lymphatic vessels. Eur. J. Nucl. Med..

[47] Thompson B., Gaitatzis K., Janse de Jonge X., Blackwell R., Koelmeyer L.A. (2021). Manual lymphatic drainage treatment for lymphedema: A systematic review of the literature. J. Cancer Surviv..

[48] Ramadan F. (2024). Manual lymphatic drainage: The evidence behind the efficacy. Br. J. Community Nurs..

[49] Zolla V., Nizamutdinova I.T., Scharf B., Clement C.C., Maejima D., Akl T., Nagai T., Luciani P., Leroux J.C., Halin C. (2015). Aging-related anatomical and biochemical changes in lymphatic collectors impair lymph transport, fluid homeostasis, and pathogen clearance. Aging Cell.

[50] Weiss M., Baumeister R.G., Hahn K. (2003). Dynamic lymph flow imaging in patients with oedema of the lower limb for evaluation of the functional outcome after autologous lymph vessel transplantation: An 8-year follow-up study. Eur. J. Nucl. Med. Mol. Imaging.

[51] Weiss M., Baumeister R.G., Hahn K. (2002). Post-therapeutic lymphedema: Scintigraphy before and after autologous lymph vessel transplantation: 8 years of long-term follow-up. Clin. Nucl. Med..

[52] Stucker M., Protz K., Eder S., Lauchli S., Traber J., Dissemond J. (2023). Diagnosis of leg edema. Dermatologie.

[53] Koban K.C., Titze V., Etzel L., Frank K., Schenck T., Giunta R. (2018). Quantitative volumetric analysis of the lower extremity: Validation against established tape measurement and water displacement. Handchir. Mikrochir. Plast. Chir..

[54] Schiltz D., Diesch S.T., Kiermeier N., Eibl D., Felmerer G., Schreml S., Biermann N., Prantl L., Taeger C.D. (2024). Digital Volumetric Measurements Based on 3D Scans of the Lower Limb: A Valid and Reproducible Method for Evaluation in Lymphedema Therapy. Ann. Vasc. Surg..

[55] Etzel L., Schenck T.L., Giunta R.E., Li Z., Xu Y., Koban K.C. (2021). Digital Leg Volume Quantification: Precision Assessment of a Novel Workflow Based on Single Capture Three-dimensional Whole-Body Surface Imaging. J. Digit. Imaging.

[56] Verhulst A.C., Wesselius T.S., Glas H.H., Vreeken R.D., Ulrich D.J.O., Maal T.J.J. (2017). Accuracy and reproducibility of a newly developed tool for volume measurements of the arm using 3D stereophotogrammetry. J. Plast. Reconstr. Aesthetic Surg..

